# Maintenance therapy in hormone receptor-positive and human epidermal growth factor receptor 2-positive metastatic breast cancer: a real-world multicenter retrospective study

**DOI:** 10.3389/fonc.2025.1703657

**Published:** 2026-01-09

**Authors:** Shengnan Bao, Jia Chen, Yongmei Yin

**Affiliations:** 1 Department of Oncology, Nantong Tumor Hospital, Affiliated Tumor Hospital of Nantong University, Nantong, China; 2 Department of Oncology, The First Affiliated Hospital of Nanjing Medical University, Nanjing, China; 3 Jiangsu Key Lab of Cancer Biomarkers, Prevention and Treatment, Collaborative Innovation Center for Personalized Cancer Medicine, Nanjing Medical University, Nanjing, China

**Keywords:** hormone receptor, human epidermal growth factor receptor 2, maintenance therapy, metastatic breast cancer, nomogram

## Abstract

**Introduction:**

The optimal choice of maintenance therapy after standard first-line treatment for hormone receptor (HR)-and human epidermal growth factor receptor 2 (HER2)-positive metastatic breast cancer (MBC) remains uncertain. Therefore, we evaluated the clinical value of trastuzumab combined with endocrine therapy (ET group) compared with trastuzumab combined with chemotherapy (CT group) as maintenance therapy in patients with HR+/HER2+ MBC.

**Methods:**

The clinical data of 117 patients with HR+/HER2+ MBC who received trastuzumab combined with endocrine therapy or chemotherapy as maintenance therapy at three hospitals in China between January 2012 and October 2022 were retrospectively analyzed. The primary endpoint was progression-free survival (PFS). To construct a nomogram, a Cox regression model was used for both the univariate and multivariate analyses. The predictive ability and accuracy of the nomogram were assessed using the concordance index and calibration curves.

**Results:**

Herein, 73 and 44 patients were assigned to the ET and CT groups, respectively. PFS was significantly longer in the ET group than the CT group (median PFS, 10.8 months vs. 7.2 months; adjusted hazard ratio, 0.68; 95% confidence interval, 0.46 to 0.99]; p=0.039). Based on the results of the multivariate analysis, a nomogram was established, which enabled visual risk prediction and demonstrated acceptable predictive ability.

**Discussion:**

Maintenance therapy using trastuzumab combined with endocrine therapy following standard first-line treatment may improve the survival and safety of patients with HR+/HER2+ MBC.

## 1 Introduction

Breast cancer with high human epidermal growth factor receptor 2 (HER2) expression has traditionally been associated with poor survival outcomes (1). Despite prolonged treatment, most patients with HER2+ metastatic breast cancer (MBC) eventually succumb to the disease (2, 3). The development of anti-HER2 therapies has significantly improved survival (4). However, many patients continue to require new treatment strategies because of the heterogeneity of HER2+ breast cancer. This heterogeneity is reflected in the simultaneous expression of hormone receptors (HRs), observed in approximately 10% of breast cancers (5, 6). The biological behavior of HR+/HER2+ breast cancer remains poorly elucidated, posing significant challenges in clinical practice.

According to the current guidelines for MBC, the standard first-line regimen for all suitable HER2+ breast cancer is chemotherapy combined with anti-HER2 therapy, regardless of the HR expression status (7–9). However, not all patients with HR+/HER2+ subtypes require or tolerate chemotherapy, for whom anti-HER2 therapy combined with endocrine therapy may be a potential alternative. Previous studies have demonstrated that complex crosstalk between HER2 and HR leads to endocrine therapy resistance (10, 11). In addition, estrogen receptors (ERs) located in or near the cell membrane can activate growth factor receptor tyrosine kinases (such as HER2), providing estrogen with another mechanism for promoting growth (12, 13). Therefore, we believe that targeting both HER2 and HR may improve patient survival in HR+/HER2+ MBC.

In a previous study, the survival of patients with MBC, including progression-free survival (PFS) and overall survival (OS), improved after extending the duration of first-line chemotherapy (14). These findings have prompted in-depth investigations into the effect of maintenance therapy on patients with MBC. After receiving standard first-line chemotherapy to achieve disease control, including complete response, partial response, and stable disease, it is necessary to increase the time of drug treatment to further improve PFS and patient tolerance.

Given the unique biological and behavioral characteristics of HR+/HER2+ MBC, no consensus regarding long-term maintenance therapy currently exists. Accordingly, we compared the efficacy of trastuzumab plus endocrine therapy and trastuzumab plus chemotherapy as maintenance therapy. Additionally, we developed a prognostic model to estimate the survival rates of individual patients more accurately, aiming to advance individualized therapy through quantitative analysis of prognostic factors.

## 2 Materials and methods

### 2.1 Patients

Herein, the clinical data of 117 patients with MBC who underwent treatment at Jiangsu Province Hospital, Henan Cancer Hospital, or Zhejiang Cancer Hospital between January 1, 2012, and October 31, 2022, were retrospectively analyzed. The primary inclusion criteria were as follows: (1) women aged ≥ 18 years with histology-confirmed MBC; (2) pathologically confirmed ER and/or progesterone receptor (PR)-positivity (≥10% positive cells in immunohistochemistry ) and HER2 - positivity (immunohistochemistry showed +++ or fluorescence in situ hybridization was positive); (3) first-line treatment with trastuzumab combined with chemotherapy to achieve disease control; (4) receiving trastuzumab combined with endocrine therapy (ET group) or chemotherapy (CT group) after the standard first-line treatment; (5) measurable lesions; (6) no potentially uncontrollable diseases; (7) Eastern Cooperative Oncology Group status of 0 to 2; (8) availability of complete clinical data.

This study was approved by the Ethics Committee of the First Affiliated Hospital of Nanjing Medical University (Jiangsu Province Hospital) (approval no. 2021-SR-357).

### 2.2 Treatments

Patients underwent maintenance therapy using trastuzumab combined with endocrine therapy (aromatase inhibitors, fulvestrant, tamoxifen, or toremifene) or trastuzumab combined with chemotherapy (capecitabine or vinorelbine). The selection of a treatment regimen is contingent upon the clinician’s expertise and the patient’s preferences. Further treatment details were presented in **Supplementary Table 1**.

Trastuzumab was administered intravenously at a dose of 6 mg/kg every 3 weeks. Tamoxifen was administered orally at a dose of 10 mg twice daily. Toremifene was administered orally at a dose of 60 mg once daily. Anastrozole, letrozole, and exemestane were administered orally at doses of 1, 2.5, or 25 mg once daily. Fulvestrant was administered intramuscularly at a dose of 500 mg every 28 days, with an additional dose administered on day 15 of cycle 1. Capecitabine was administered orally at a dose of 1,250 mg/m^2^ twice daily for 2 weeks, followed by a 1-week rest period, in 3-week cycles. Regarding vinorelbine, 60 mg was administered orally in the first week; if there were no obvious side effects, the dose was increased to 80 mg every 3 weeks.

### 2.3 Outcomes

Patients were evaluated according to the Response Evaluation Criteria in Solid Tumors (RECIST v1.1) guidelines (15). The primary endpoint was PFS, defined as the interval between the start of maintenance therapy and tumor progression or patient death. Adverse events were classified according to the National Cancer Institute Common Terminology Criteria for Adverse Events version 4.0.

### 2.4 Statistical analysis

The chi-square test was used to compare categorical clinical characteristics between the groups. Survival outcomes were estimated using Kaplan-Meier analysis, and differences in PFS stratified covariates were evaluated using the log-rank test. Hazard ratio (HR) and 95% confidence intervals (CIs) for potential prognostic factors were estimated using univariate and multivariate Cox proportional hazard regression models. Subgroup differences in PFS were expressed using forest plots. Variables with p < 0.2 in univariate analysis were included in the multivariate analysis, and a nomogram capable of visual risk prediction was established. To assess the prediction accuracy of the nomogram, the concordance index (C-index) was calculated using the bootstrap method with 100 iterations, and calibration curves were generated. The C-index, with a high prediction accuracy in the range of 0.5-1.0, reflects the discriminative ability of the nomogram. Conversely, calibration curves assess the accuracy of the model by evaluating the agreement between the observed and predicted PFS, with close alignment with the diagonal indicating better accuracy. Data were analyzed using SPSS (version 23.0) and R (version 4.0.3) software. A two-tailed p < 0.05 was considered statistically significant.

## 3 Results

### 3.1 Patient characteristics

In the ET group, trastuzumab combined with endocrine therapies, such as fulvestrant, tamoxifen, toremifene, or aromatase inhibitors, was administered to 73 patients (62.4%). In the CT group, 44 (43.6%) patients received trastuzumab in combination with capecitabine or vinorelbine. The median patient age was 48 (range, 25-83) years. Herein, 43 women were postmenopausal and 61 were premenopausal. Fifteen patients were newly diagnosed with stage IV disease, and 84 had recurrent and metastatic disease. Additionally, 81 patients received adjuvant endocrine therapy, and 42 received adjuvant radiotherapy. The baseline characteristics did not differ significantly between the groups (**Table 1**).

**Table 1:** Patient characteristics at baseline.

Variable	ET group (n = 73)	CT group (n = 44)	*P* value
Age (year)			0.867
< 50	38 (52.1%)	22 (50.0%)	
≥ 50	33 (45.2%)	20 (45.5%)	
Unknown	2 (2.7%)	2 (4.5%)	
Menopausal status			0.741
Postmenopausal	25 (34.2%)	18 (40.9%)	
Premenopausal	40 (54.8%)	21 (47.7%)	
Unknown	8 (11.0%)	5 (11.4%)	
Pathologic tumor stage			0.491
Stage I-III	54 (74.0%)	30 (68.2%)	
Stage IV	10 (13.7%)	5 (11.4%)	
Unknown	9 (12.3%)	9 (20.5%)	
Number of metastatic sites			0.612
1	30 (41.1%)	16 (36.4%)	
≥ 2	43 (58.9%)	28 (63.6%)	
Metastases			
Brain	9 (12.3%)	6 (13.6%)	0.838
Bone	33 (45.2%)	18 (40.9%)	0.650
Viscera (liver and/or lung)	41 (56.2%)	31 (70.5%)	0.124
Other	50 (68.5%)	26 (59.1%)	0.302
Adjuvant endocrine therapy			0.849
Yes	51 (69.9%)	30 (68.2%)	
No	22 (30.1%)	14 (31.8%)	
Adjuvant radiotherapy			0.202
Yes	23 (31.5%)	19 (43.2%)	
No	50 (68.5%)	25 (56.8%)	

*ET*, trastuzumab combined with endocrine therapy; *CT*, trastuzumab combined with chemotherapy.

### 3.2 Efficacy

**Figure 1:**
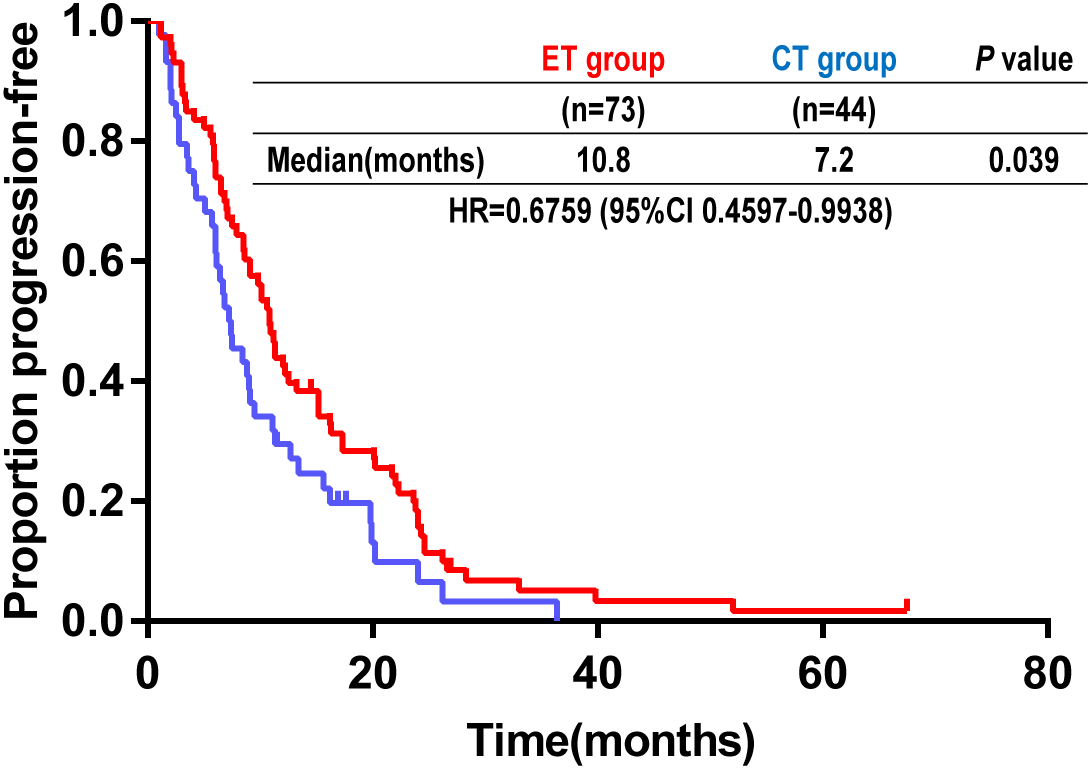
Kaplan-Meier analysis of progression-free survival in all patients. ET, trastuzumab combined with endocrine therapy; CT, trastuzumab combined with chemotherapy; HR, hazard ratio; CI, confidence interval.

The median PFS was 10.8 (95% CI, 9.4-12.2) months in the ET group compared with 7.2 (5.0-9.4) months in the CT group (hazard ratio, 0.68; 95% CI, 0.46-0.99; *p* = 0.039; **Figure 1**). In the univariate analysis based on the Cox regression model, age, menopausal status, newly diagnosed clinical stage, number of metastases, metastatic location, adjuvant endocrine therapy, and adjuvant radiotherapy were not significantly associated with PFS (**Table 2**). Age, menopausal status, metastatic location, and maintenance therapy were further analyzed in a multivariate Cox regression analysis. Multivariate analysis revealed that maintenance therapy was an independent predictor of PFS in patients with HR+/HER2+ MBC (**Table 2**).

**Table 2:** Univariate and multivariate analysis based on Cox regression model.

Risk factors	Univariate analysis		Multivariate analysis	
	HR (95%CI)	*P* value	HR (95%CI)	*P* value
Age (year)				
< 50	reference		reference	
≥ 50	0.71 (0.49-1.04)	0.082	0.73 (0.45-1.20)	0.214
Unknown	0.26 (0.06-1.08)	0.063		
Menopausal status				
Postmenopausal	reference		reference	
Premenopausal	1.34 (0.89-2.01)	0.163	1.14 (0.68-1.89)	0.624
Unknown	0.79 (0.42-1.48)	0.456		
Pathologic tumor stage				
Stage I-III	reference			
Stage IV	1.00 (0.58-1.75)	0.990		
Unknown	1.20 (0.72-2.01)	0.486		
Number of metastatic sites				
1	reference			
≥ 2	0.99 (0.67-1.46)	0.948		
Metastases				
Brain	1.18 (0.68-2.05)	0.559		
Bone	0.74 (0.51-1.09)	0.127	0.86 (0.58-1.27)	0.443
Viscera	0.96 (0.65-1.42)	0.845		
Other	1.07 (0.72-1.58)	0.749		
Adjuvant endocrine therapy				
Yes	reference			
No	0.83 (0.55-1.24)	0.354		
Adjuvant radiotherapy				
Yes	reference			
No	0.81 (0.54-1.19)	0.277		
Treatment				
CT	reference		reference	
ET	0.66 (0.45-0.98)	0.039	0.57 (0.38-0.85)	0.006

*HR*, hazard ratio; *CI*, confidence interval; *ET*, trastuzumab combined with endocrine therapy; *CT*, trastuzumab combined with chemotherapy.

**Figure 2:**
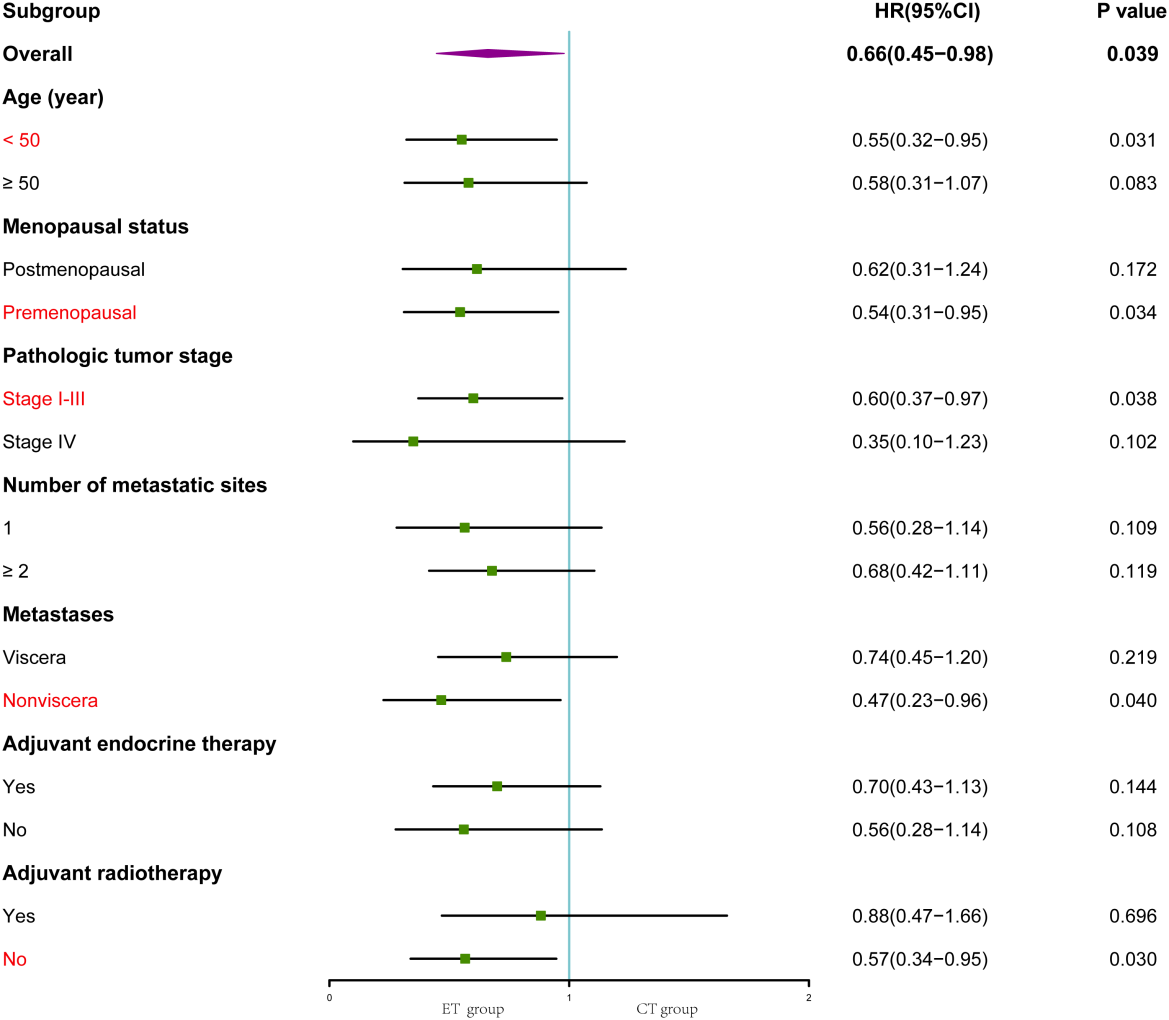
Forest plot of subgroup analysis. Subgroups with P<0.05 are indicated in red font. HR, hazard ratio; CI, confidence interval; ET, trastuzumab combined with endocrine therapy; CT, trastuzumab combined with chemotherapy.

Among all analyzed subgroups, trastuzumab combined with endocrine therapy demonstrated better efficacy in patients who were younger than 50 years (hazard ratio, 0.55; 95% CI, 0.32-0.95; *p* = 0.031), premenopausal (hazard ratio, 0.54; 95% CI, 0.31-0.95; *p* = 0.034), newly diagnosed with non-stage IV disease (hazard ratio, 0.60; 95% CI, 0.37-0.97; *p* = 0.038), without visceral metastasis (hazard ratio, 0.47; 95% CI, 0.23-0.96; *p* = 0.040), and without adjuvant radiotherapy (hazard ratio, 0.57; 95% CI, 0.34-0.95; *p* = 0.030) (p < 0.05; **Figure 2**).

### 3.3 Development and validation of the prognostic nomogram

**Figure 3:**
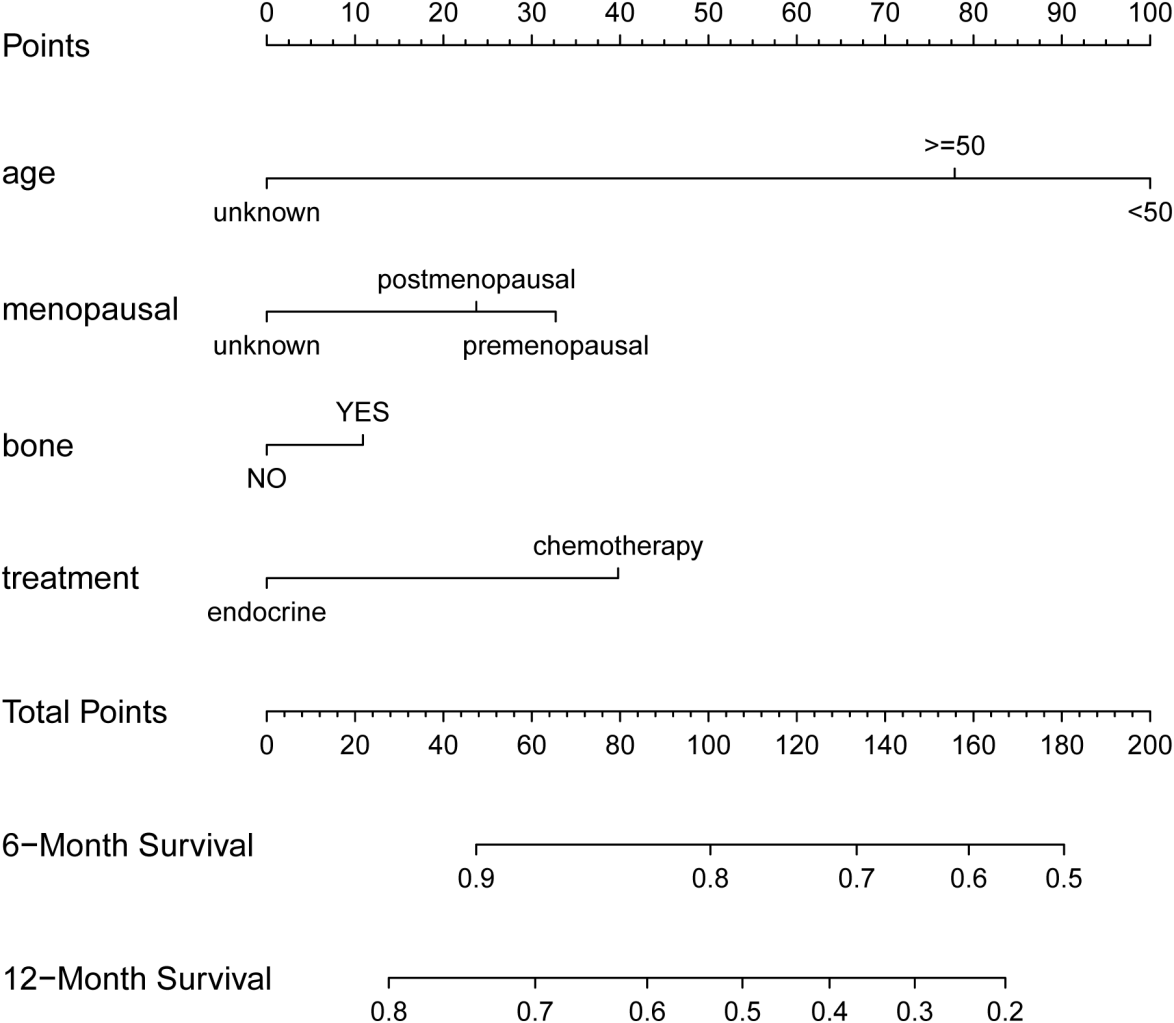
Prognostic nomogram of progression-free survival.

**Figure 4:**
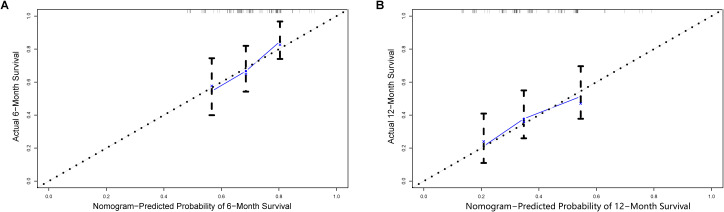
Validation of the nomogram. Calibration curves for 6-month **(A)** and 12-month **(B)** progression-free survival probabilities.

As shown in **Figure 3**, age exerted the greatest influence on prognosis, followed by maintenance therapy, menopausal status, and bone metastasis. The different states for each clinical variable correspond to different points on the integral scale. We estimated the probability of 6-month and 12-month PFS in patients with HR+/HER2+ MBC by calculating the sum of each item’s score. The C-index for the internal validation of the nomogram was 0.628 (95% CI, 0.577-0.679). The corresponding calibration curves for 6-month and 12-month PFS were closely aligned with the reference line (**Figure 4**), indicating high reliability.

### 3.4 Safety

**Table 3** presents the safety profile observed herein. In the ET group, high rates of arthralgia (12.3%), nausea (9.6%), and diarrhea (9.6%) were observed, all of which were grade 1–2 adverse events; no adverse events of grade ≥3 were observed. In the CT group, major adverse events included hand-foot syndrome (70.5%), diarrhea (56.8%), and nausea (43.2%); grade ≥3 adverse events included hand-foot syndrome (20.5%), diarrhea (6.8%), neutropenia (6.8%), and nausea (2.3%).

**Table 3:** Treatment-related adverse events.

Events	ET group (n = 73)		CT group (n = 44)	
Adverse Event	All grades	Grade ≥ 3	All grades	Grade ≥ 3
Neutropenia	4(5.5%)	0	7(15.9%)	3(6.8%)
Diarrhea	7(9.6%)	0	25(56.8%)	3(6.8%)
Nausea	7(9.6%)	0	19(43.2%)	1(2.3%)
Hand-foot syndrome	1(1.4%)	0	31(70.5%)	9(20.5%)
Rash	1(1.4%)	0	3(6.8%)	0
Arthralgia	9(12.3%)	0	3(6.8%)	0

*ET*, trastuzumab combined with endocrine therapy; *CT*, trastuzumab combined with chemotherapy.

## 4 Discussion

Over the past three decades, the treatment and understanding of HER2+ breast cancer have advanced substantially, leading to improved survival rates in MBC. Distinct biological differences exist between HR+/HER2+ and HR-/HER2+ breast cancers. In HR+/HER2+ breast cancer, endocrine and anti-HER2 therapies may be affected by crosstalk between ER and HER2 pathways (16). Subgroup analyses of trials involving HER2+ breast cancer patients, as well as clinical trials specifically designed to identify the optimal treatment strategy for HR+/HER2+ breast cancer, can help guide treatment decisions (17, 18). However, careful trial designing is required to account for the heterogeneity of breast cancer. To date, drug development has generally targeted either ER or HER2, which are considered the two most important factors associated with tumor growth and survival in breast cancer. However, the complex connections between ER and HER2 signaling pathways, as well as other well-known therapeutic targets, are well known.

SystHERs (NCT01615068) is a prospective, multicenter, observational cohort study conducted in the United States (19). Therein, of 977 patients with HER2+ MBC, 685 were HR+ and 292 were HR-. In the HR+ cohort, the median OS and PFS were longer in patients who received first-line endocrine therapy compared with those who did not. In another similar prospective observational registry study, dual-targeting of HR and HER2 yielded better outcomes compared with anti-HER2 therapy alone in patients with HR+/HER2+ MBC, irrespective of concurrent chemotherapy (20). In the recently reported multicenter, phase III, randomized controlled trial SYSUCC-002 in China, trastuzumab combined with endocrine therapy as a first-line regimen in HR+/HER2+ MBC was not inferior to trastuzumab combined with chemotherapy, with the additional benefit of less severe side effects (median PFS, 19.2 months vs. 14.8 months; HR, 0.88 [95% CI, 0.71 to 1.09]; P_noninferiority_ <0.0001) (21).

To date, no prospective randomized controlled trials have investigated the efficacy of maintenance therapy after standard first-line treatment in HR+/HER2+ MBC. In clinical practice, combination treatment using trastuzumab and endocrine therapy or chemotherapy remains an alternative (22). Herein, the median PFS in the ET group was 10.8 months, and the median PFS in the CT group was 7.2 months (HR = 0.68; 95%CI, 0.46-0.99; *p* = 0.039), suggesting that this “de-chemotherapy” regimen was effective and more tolerable. Endocrine therapy may be more suited to patients with contraindications to chemotherapy, those unwilling to undergo chemotherapy, and those with low disease burden and high HR expression. Preclinical evidence indicates that interaction between HER2 and HR signaling pathways can lead to endocrine resistance. Thus, concurrently blocking HER2 and HR pathways could provide therapeutic benefit (23).

Notably, in the ET group, patients who were younger than 50 years, premenopausal, newly diagnosed with non-stage IV disease, without visceral metastasis, and without adjuvant radiotherapy exhibited better treatment efficacy. We hypothesize that this group of likely low-risk patients may benefit more from the “de-chemotherapy” program. Furthermore, the intrinsic subtype determined by Prediction Analysis of Microarray 50 (PAM50) serves as a basis for further patient stratification, which may yield predictive information regarding chemotherapy outcomes (24). This observed therapeutic heterogeneity requires further investigation, which should incorporate adaptive study design, biomarker identification, and precise subgroup stratification.

The safety data from our study did not reveal any new safety signals. Consistent with previous trials (18, 25), arthralgia, nausea, and diarrhea were common, predominantly low-grade adverse events in the ET group. However, in clinical practice, hand-foot syndrome induced by capecitabine represents a dose-limiting toxicity that leads to poor tolerance in a substantial proportion of patients. Therefore, the endocrine therapy arm demonstrated a more favorable safety profile.

Our study has several strengths. First, we collected clinical data from multiple institutions, providing practical insights into guiding treatment decisions in the Chinese population. Additionally, to our knowledge, this is the first study to construct a nomogram for predicting survival in patients with HR+/HER2+ MBC. The model showed acceptable discrimination, though this value indicated room for improvement in risk stratification. Importantly, the calibration curve demonstrated good agreement between predicted and observed outcomes. Therefore, while the model’s discriminative power was modest, it may still offer clinically useful probability estimates for individual patients when combined with clinical judgment. These preliminary findings may help clinicians make better clinical decisions in the management of patients with HR+/HER2+ MBC.

Our study has some limitations. First, although dual HER2 blockade with trastuzumab and pertuzumab is currently the first-line anti-HER2 therapy (26), recent data from the DESTINY-Breast09 trial indicate that trastuzumab deruxtecan (T-DXd) combined with pertuzumab may redefine first-line therapy by offering enhanced survival benefits. Future studies should integrate these regimens to improve population outcomes. With the ongoing development of new drugs, treatment recommendations are constantly evolving. However, the clinical concepts put forward by these classical regimens still have significant implications. Second, the sample size was small, and the number of patients in the two groups was not balanced. Finally, considering the limitations of this retrospective study, further prospective analyses with larger cohorts are warranted.

In conclusion, maintenance therapy using trastuzumab combined with endocrine therapy after standard first-line treatment can improve the survival and safety of patients with HR+/HER2+ MBC. The developed nomogram is simple, has moderate clinical applicability, and demonstrates good correction ability and acceptable discriminative power. It can be used to estimate the prognosis of patients with breast cancer and develop individualized therapeutic strategies.

## Data Availability

The original contributions presented in the study are included in the article/**Supplementary Material**. Further inquiries can be directed to the corresponding authors.

## Supplementary material

Supplementary Table 1 Details of therapeutic regimens for the study patients.
